# Pharmacological fMRI provides evidence for opioidergic modulation of discrimination of facial pain expressions

**DOI:** 10.1111/psyp.13717

**Published:** 2020-11-03

**Authors:** Yili Zhao, Markus Rütgen, Lei Zhang, Claus Lamm

**Affiliations:** ^1^ Social, Cognitive and Affective Neuroscience Unit Department of Cognition, Emotion, and Methods in Psychology Faculty of Psychology University of Vienna Vienna Austria; ^2^ Vienna Cognitive Science Hub University of Vienna Vienna Austria; ^3^ Neuropsychopharmacology and Biopsychology Unit Department of Cognition, Emotion, and Methods in Psychology Faculty of Psychology University of Vienna Vienna Austria

**Keywords:** emotion discrimination, facial expression, fMRI, naltrexone, opioid system, pain, psychopharmacology, right fusiform face area

## Abstract

The endogenous opioid system is strongly involved in the modulation of pain. However, the potential role of this system in perceiving painful facial expressions from others has not been sufficiently explored as of yet. To elucidate the contribution of the opioid system to the perception of painful facial expressions, we conducted a double‐blind, within‐subjects pharmacological functional magnetic resonance imaging (fMRI) study, in which 42 participants engaged in an emotion discrimination task (pain vs. disgust expressions) in two experimental sessions, receiving either the opioid receptor antagonist naltrexone or an inert substance (placebo). On the behavioral level, participants less frequently judged an expression as pain under naltrexone as compared to placebo. On the neural level, parametric modulation of activation in the (putative) right fusiform face area (FFA), which was correlated with increased pain intensity, was higher under naltrexone than placebo. Regression analyses revealed that brain activity in the right FFA significantly predicted behavioral performance in disambiguating pain from disgust, both under naltrexone and placebo. These findings suggest that reducing opioid system activity decreased participants' sensitivity for facial expressions of pain, and that this was linked to possibly compensatory engagement of processes related to visual perception, rather than to higher level affective processes, and pain regulation.

## Abstract 2

The behavioral and neural findings of this psychopharmacological fMRI study shed light on a causal role of the opioid system in the discrimination of painful facial expressions, paving the way for further exploration of clinical implications in the domains of pain diagnosis and treatment, on the one hand, and future research on the relationship between basic socio‐perceptual processing and empathy, on the other.

## 1 INTRODUCTION

The ability to perceive pain in others is fundamental in social interaction, as it strengthens social connections and promotes care for others' well‐being. Studies have shown that when observing others in pain, brain regions involved in the processing of self‐directed pain were also activated, but this mainly included parts of the affective‐motivational component of pain processing, such as the anterior mid‐cingulate cortex (aMCC) and the anterior insula (AI) (Botvinick et al., 2005; Lamm et al., 2011; Singer et al., 2004). Recent meta‐analytic research has revealed additional activations shared by empathy for pain and self‐direct pain in the inferior frontal gyrus and supramarginal gyri (Fallon et al., 2020). Besides, another meta‐analysis research demonstrated that the core neural empathy network (aMCC and AI) was activated when observing others in painful states as well as in nonpain negative affective states (Timmers et al., 2018). Pain is commonly conveyed to others via facial expressions. In those studies where visual perception of other's facial expressions of pain was critical to recognize their emotional state, the visual cortex, especially the fusiform face area (FFA), has also been repeatedly found as activated (Botvinick et al., 2005; Decety et al., 2013; Lamm et al., 2007; Simon et al., 2006). Recent meta‐analyses have reported involvement of the fusiform gyrus (along with aMCC and AI) in empathy paradigms employing facial expressions (Jauniaux et al., 2019; Xiong et al., 2019).

At the neurochemical level, the endogenous opioid system has been recognized to play an essential role in the experience of pain. Abundant studies have demonstrated that the opioid system is involved in the modulation of self‐pain experience in both sensory and affective states (Botvinick et al., 2005; Lamm et al., 2011; Singer et al., 2004). Recently, research has reported that opioid antagonists (e.g., naltrexone) and agonists (e.g., buprenorphine) could alter individuals' sensitivity or responses to facial expressions of emotions such as anger, fear, happiness, or sadness, in others (Ipser et al., 2013; Meier et al., 2016; Wardle et al., 2016). However, the findings of these studies were rather inconsistent, suggesting that definite conclusions regarding the role of the opioid system in recognizing the emotions of others are still not possible.

Moreover, mechanistic evidence on whether and how the endogenous opioid system functions when perceiving others' pain is still lacking. Using positron emission tomography, Karjalainen et al. (2017) revealed the involvement of μ‐opioid receptor in vicarious pain, and psychopharmacological and neuroimaging findings of our own lab (Rütgen et al., 2015a, 2015b, 2018) indicate a causal role of the opioid system in empathy for pain. Recent mediation analyses, though somewhat inconclusively, suggest that this may be explained by effects on emotion identification (Coll et al., 2017), while related research revealed that other neurochemical mechanisms might play a role in empathy for pain as well (Mischkowski et al., 2016). However, since none of these studies had a specific focus on the visual recognition and processing of others' facial pain expressions, it remains unclear whether there is a specific relationship between the opioid system and the perception of pain expressions.

To bridge this gap, we adopted an emotion discrimination paradigm (Cook et al., 2013; Young et al., 2002), to investigate whether the opioid system influences discrimination of morphed facial expressions between pain and another emotion (i.e., disgust in the present study). The reasons to morph disgust, instead of other emotions, with pain were twofold: (a) Pain and disgust facial expressions share some similarities but are still distinct enough to be distinguished from each other (Kunz et al., 2013; Sharvit et al., 2015); (b) According to a recent review (Nummenmaa & Tuominen, 2018), the endogenous opioid system is engaged in modulating a wide range of basic emotions (e.g., anger, fear, sadness, and pleasure), while there is only scarce evidence for the potential involvement of the opioid system in the modulation of disgust (which appears to be rather susceptible to other types of modulation, such as the oxytocin system; see Kavaliers et al., 2019). Disgust for these two main reasons thus appeared to be a reasonable choice, especially in comparison to other emotions. However, when designing the study and when discussing the results, we were aware that absence of evidence does not imply evidence of absence of effects on disgust.

Specifically, in a pharmaco‐functional magnetic resonance imaging (fMRI) study, we applied an emotion discrimination task to examine whether administration of the opioid antagonist naltrexone influenced how painful facial expressions were discriminated from disgust expressions, and to which brain areas this was associated. In terms of our initial research interest, the focus was on the core empathy affective regions (i.e., aMCC and bilateral AI) and regions of interests (ROIs) were determined accordingly (based on the meta‐analysis of Lamm et al., 2011; see also Rütgen et al., 2015). Besides, since results from the parametric modulation analysis showed significant activity in the FFA (especially in the right hemisphere), we identified this region as a fourth (post‐hoc exploratory) ROI. Apart from FFA, the superior temporal sulcus (STS) is another region that was frequently reported in studies of facial expression processing (Engell & Haxby, 2007; Narumoto et al., 2001; Wegrzyn et al., 2015). However, we did not find significant parametric modulation of activation in the STS. Studies have shown that the STS is preferentially engaged during the processing of dynamic facial expressions; the FFA, on the contrary, is preferentially engaged during the processing of static facial expressions (De Winter et al., 2015; Pitcher et al., 2011). Given that only static facial expressions were used in the present study, this may be the reason why we only saw parametric modulation in FFA, which is why we focused on this area rather than STS. Based on the rather controversial previous findings, our hypothesis regarding behavioral discrimination was two‐sided, while our analyses of the neural underpinnings focused on areas related to the discrimination of pain expressions, including the FFA, the anterior mid‐cingulate cortex (aMCC), and the anterior insular cortex (AI).

## 2 METHOD

### 2.1 Participants

Fifty‐two participants (30 females; age: 24.06 ± 3.39 years) were recruited through online advertisements. Exclusion criteria were left‐handedness and any history or presence of neurological and psychiatric disorders. In addition to the online MRI safety‐check questionnaire, all participants were screened by a physician at the Faculty of Psychology, University of Vienna, including a medical history check, and basic physical examination. Nine participants who did not show up for the second session were excluded. One further exclusion was due to a consistent failure to discriminate pain and disgust expressions. The final data set thus consisted of 42 participants (24 females; age: 24.12 ± 3.50 years). The study was approved by the ethics committee of the Medical University of Vienna and was conducted in line with the latest version of the Declaration of Helsinki (2013) of the World Medical Association (https://www.wma.net/policies‐post/wma‐declaration‐of‐helsinki‐ethical‐principles‐for‐medical‐research‐involving‐human‐subjects/). All participants provided written consent to participate.

### 2.2 Paradigm

**FIGURE 1:**
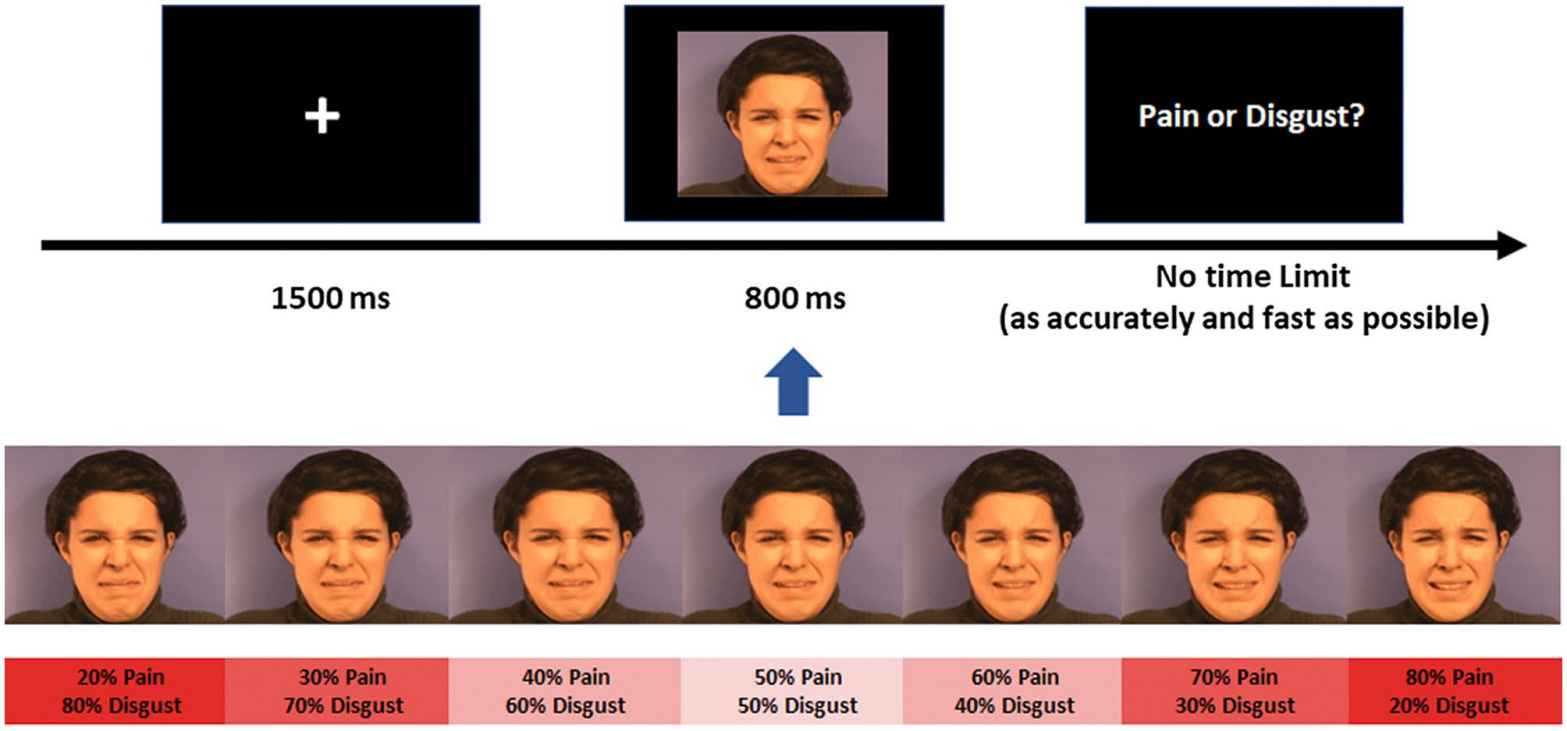
Experimental procedure. Upper panel: Following a fixation cross (displayed for 1,500 ms), participants were presented with a morphed facial expression image selected from one out of seven options differing in the composition of pain and disgust intensities (e.g., 50% pain and 50% disgust, as shown here) lasting for 800 ms. After each stimulus, participants needed to judge whether the presented expression was pain or disgust. Lower panel: The facial expressions continuum from one of the two female characters. The seven stimuli are shown here for illustration purposes, participants only saw the images in the upper row, at full‐screen size

While their brains were being scanned using fMRI, participants were engaged in a modified version of the emotion discrimination task (Cook et al., 2013), in which facial expressions were morphed along a continuum between pain and disgust from 20% to 80% in 10% steps. In the original version of Cook et al., a surprise‐fear continuum was derived from the same person, comprising seven images morphing between surprise and fear while holding the face identity constant. Here, we adopted a pain‐disgust continuum using pictures of pain and disgust expressions from the same person. In each trial, after 1,500 ms fixation, a facial expression was presented on the screen, which consisted of a morph between two original images showing pain and disgust (e.g., 50% pain and 50% disgust) for 800 ms. The stimulus presentation time was chosen according to the one of the original task paradigm by Cook and colleagues (2013). Following each stimulus, a prompt asking “pain or disgust?” was presented on the screen, and participants were required to judge whether the previous expression was showing pain, or disgust (see Figure 1). Studies have found that the gender of targets affected observer's speed and accuracy as well as neural responses when detecting facial expressions of pain (Riva et al., 2011; Simon et al., 2006), which seemed to suggest a potential difference in the mechanism underlying processing pain expressions of male and female targets. Consequently, we decided to use faces from one gender (female here) to minimize this potential confound of the behavioral and neural responses. The original facial expressions of pain and disgust had been extracted from the Montreal Pain and Affective Face Clips (Simon et al., 2008), using images extracted from two females' clips. Morphs were adopted and generated with FantaMorph 5 (Deluxe edition; http://www.fantamorph.com/), with each individual morph image using pain and disgust pictures from the same female.

### 2.3 Procedure

Participants were invited to visit the lab twice to take part in two fMRI sessions, separated by at least one week, to ensure complete drug washout (Bisaga et al., 2018). In each session, participants received either 50 mg naltrexone or an inert substance (i.e., placebo), in a double‐blind fashion. Session order was counterbalanced, and pills were delivered with the cover story that they were “MRI signal enhancer pills,” which was done in order to avoid that subjective beliefs about the study aims and opioid action would bias the results. However, participants were fully informed about the possible effects in the consent form, including possible side effects of naltrexone as part of the consent form. All participants signed the consent form as an agreement of participation after they completely understood and already evaluated any possible risk or adverse effects regarding the naltrexone administration.

Experimental sessions took place at the University of Vienna MR Centre. Before administering the pill, participants were screened for drug consumption again, using a urinary drug test. Then, participants were instructed to orally take a pill, which either contained naltrexone or the inert substance (placebo). They were further required to wait for 45 min for the drug to take effect (KatzenPerez et al., 2001; Price et al., 2016). After the waiting time, participants experienced a cold pressor test (CPT), in which they were asked to immerse one of their hands into cold water (1~5°C) as long as they could. The CPT procedure could promote endogenous opiate activation (Jungkunz et al., 1983; Robertson et al., 2008; Washington et al., 2000). From an experimental design perspective, we feared that the effect of the opioid blockade would be negligible at a baseline level. By applying a cold pressor test (CPT), we sought to induce endorphin release (Casale et al., 1985; King et al., 2013) and, consequently, a greater difference between placebo and naltrexone sessions. Afterward, participants were led into the scanner room and first underwent an empathy for pain task (Rütgen et al., 2015b), whose findings are outside the scope of the present paper, and then, the emotion discrimination task. In the emotion discrimination task, participants were required to judge 140 morphed facial expressions presented in a pseudorandom order on whether it was pain or disgust, by pressing either the left or right button on the MRI‐compatible button box. The left button represented the choice of pain expression, and the right button represented the choice of disgust expression. This button assignment was kept identical to the original version. Participants were instructed to respond as accurately and fast as possible. Though there was no time limit in the judgment phase, trials whose reaction time was longer than 4s were regarded as invalid, and the ratio of invalid trials was taken into account during data analysis. According to the histogram of reaction times (RT) we plotted, the bulk of RTs were below 4 s, and the RTs above 4 s were rather variable (RT range: 4.06~24.35 s). To reduce unsystematic RT variations, we excluded RTs above 4 s (3.5% of all data). No difference of trial numbers between sessions was found after excluding outliers, *t*_41_ = 1.07, *p* = .293. The jitter between trials varied at random between 3,000 and 5,000 ms (see Figure 1). Following this run, an anatomical scan was performed. After scanning, participants reported on 51 potential side‐effects of naltrexone in a binary fashion (yes/no).

Participants were scheduled for the second fMRI session at about the same time of day, but at minimum one week later. The procedure of the second session was the same as the first one, except that participants who received naltrexone in the first session were given the placebo in the second session and *vice versa*. At the end of the second session, participants were debriefed and received 90 EUR overall for their participation.

### 2.4 Behavioral analysis

We examined whether naltrexone affected participants' discrimination performance of pain expressions by comparing the fitted trends of participants' pain choices at each pain intensity. According to previous studies, the relationship between categorical perception and the morphed stimuli could be fitted into a sigmoid function (Granato et al., 2012; McCullough & Emmorey, 2009; McKone et al., 2001). Following this procedure, we fitted a sigmoid model to each participant's pain choices (proportion of answering “pain”) at seven intensities, and then, extracted the fitted parameters: the slope of the sigmoid curve and the point of subjective equivalence (PSE) at which participants equally chose pain or disgust (Cook et al., 2013) using the Palamedes toolbox (http://www.palamedestoolbox.org/). Two‐tailed paired *t*‐tests were conducted to test drug effects in slope and PSE values.

We further tested whether, on average and on any pain intensity, there were drug effects on the proportion of pain choices across the seven intensities regardless of the slope. A linear mixed effect (LME) model (M1) with drug (Naltrexone vs. Placebo), pain intensity (20% to 80%), and their interaction as fixed factors and subject identity as the random intercept was created. Subject identity here refers to the identifiers (i.e., subject ID) that were used to encode and discriminate different subjects, and they were applied as random intercepts in LMEs. To set subject identity as random intercepts could effectively control the inter‐subject variation merely related to sample selection itself instead of the experimental manipulation. This approach has more advantages than the traditional ANOVA method. As the interaction was not significant, it was removed from M1. To test whether there was an alternative model that better fitted the data, we estimated a second LME model (M2) with pain intensities as random slope and performed model comparison between M1 and M2. We did not include drug groups as another random slope because it is commonly not recommended to consider as random slopes when a factor only has two levels (Barr et al., 2013). In fact, this full model, pain choices ~ drug * pain intensity + (drug * pain intensity | subject identity) failed to converge. Thus, we compared M1 and M2, and results showed that M1 (AIC: 2,641.7) better accounted for the data than M2 (AIC: 2,650.4; *χ* ^2^ = 45.33, *p* = .015). Therefore, the final LME model we chose included the two main effects of drug effect and pain intensity as fixed factors and subject identity as a random intercept, without any random slope.

Two additional LME models were constructed to test whether brain activation in the visual region of interest (ROI, see below) could predict behavioral responses, and whether this differed between the naltrexone and the placebo session. For each session, a model with the proportion of pain choices at each pain intensity as the dependent variable, percent signal change (PSC) of the seven pain intensities in the visual ROI in the corresponding session as the fixed factor, and subject identity as a random intercept was set up. Fisher's *z* transformation was performed on the coefficient of determination (*R* ^2^) of the two sessions. Based on the transformed *z* scores, a one‐sample *t* test was conducted to assess drug effects. Statistical significance was calculated with Satterthwaite approximation for degrees of freedom and set as *p* < .05.

Lastly, we tested for differences in reported side‐effects between sessions. A two‐tailed paired *t* test was performed using SPSS version 25 (IBM Corp, Armonk, NY, USA) on the sum score of all items. As nausea, one of the known potential side‐effects of naltrexone, may likely interfere with the processing of disgust, we additionally conducted a two‐tailed Fisher's exact test for this specific item.

### 2.5 MRI acquisition and data preprocessing

MRI data were acquired using a 3T Siemens Magnetom Skyra MRI scanner (Siemens, Erlangen, Germany) with a 32‐channel head coil. Functional whole‐brain scans were collected using a multiband accelerated T2*‐weighted echoplanar imaging (EPI) sequence (32 slices, multiband acceleration factor = 4, TR = 704 ms, TE = 34 ms, flip angle = 50°, FOV = 210 × 210 mm, voxel size = 2.2 × 2.2 × 3.5 mm). Structural images were acquired with a magnetization‐prepared rapid gradient‐echo (MPRAGE) sequence (176 slices, TR = 2,300 ms, TE = 2.29 ms, flip angle = 8°, voxel size = 0.9 × 0.9 × 0.9 mm, FOV = 240 × 240 mm). Imaging data were preprocessed with Statistical Parametric Mapping (SPM12; Wellcome Trust Centre for Neuroimaging, London, UK, https://www.fil.ion.ucl.ac.uk/spm/software/spm12/).

Preprocessing included realignment to the first image of the first session for both sessions, co‐registration to the T1 image, segmentation, normalization to MNI template space using Diffeomorphic Anatomical Registration Through Exponentiated Lie Algebra (DARTEL) toolbox (Ashburner, 2007), and smoothing with a 6 mm full width at half‐maximum (FWHM) Gaussian kernel.

In order to improve data quality, functional scans were individually scrubbed with the frame‐wise displacement (FD) over 0.5 mm (Power et al., 2012, 2014). That is, we identified individual outlier scans and flagged the volume indices as nuisance regressors into the General Linear Model (GLM) of the first‐level analysis.

Functional scans corresponding to trials whose reaction time was less than 100 ms or more than 4 s were identified as additional nuisance regressors (i.e., invalid trials; 7.1% of all trials, number of remaining trials (Mean ± *SD*) out of 140 trials: naltrexone session: 128.10 ± 15.97, placebo session: 132.05 ± 9.38, no significant difference between two sessions on average, *t*_41_ = −1.693, *p* = .98).

### 2.6 First‐level analysis

Two design matrices were created. First, we aimed to ensure that our task widely activated the brain network underlying perception of facial expressions as a task manipulation check (GLM1). The following regressors were entered in the model for both sessions: picture onsets of valid trials, picture onsets of invalid trials (invalid as defined above, i.e., those trials whose reaction time <100 ms or >4 s; if any), judgment onsets of valid trials, and judgment onsets of invalid trials (if any). Six head motion parameters and the scrubbing regressors (FD > 0.5 mm; see above) were further entered as nuisance regressors. A contrast (pictures > baseline, across naltrexone and placebo sessions) was created out of our main interest; besides, a reversed contrast (baseline > pictures, across naltrexone and placebo sessions) was created as a comparison to the contrast of interest. Second, we sought to test wherein the brain showed parametric responses with pain intensity of and in the morphs (i.e., 20%, 30%, 40%, 50%, 60%, 70%, and 80%; GLM2). GLM2 included the following regressors: picture onsets of valid trials, pain intensity of valid trials (parametric modulator), picture onsets of invalid trials (if any), pain intensity of invalid trials (if any), judgment onsets of valid trials, and judgment onsets of invalid trials (if any). Six head motion parameters and the scrubbing regressors (FD > 0.5 mm; see above) were further entered as nuisance regressors. Note that in GLM2, we only focused on the picture onset regressor and the pain intensity parametric regressor; all the other regressors were included to account for variances of no interest. Although no jitter was implemented between imaging viewing and the judgment phase, no multicollinearity was observed between picture onsets and the parametric regressor of pain intensities (Spearman rank‐order correlation: *r* = −.03, *p* = .77). Furthermore, the naltrexone and placebo sessions of each subject were entered separately into the same first‐level GLMs. Contrasts (i.e., naltrexone: parametric modulator > baseline; placebo: parametric modulator > baseline) were generated to assess the effect of the increased pain intensities in each session against the implicit baseline.

### 2.7 Second‐level analysis

On the group level, we implemented random‐effects analyses across all subjects in SPM12. For GLM1, the threshold for the manipulation check was set at a whole‐brain family‐wise error (FWE) correction of *p* <  .05, at the voxel level. For GLM2, we applied an initial threshold of *p* < .001 and FWE correction (*p* <  .05) at the cluster level, which is a conventional threshold in general for fMRI analyses (Woo et al., 2014). The reason for applying an even stricter threshold for the former was that GLM1 was a manipulation check (i.e., pictures > baseline), in which very strong activation, and thus, higher effect sizes were expected. The extent threshold of GLM2 was determined by the SPM extension “cp_cluster_Pthresh.m” (https://goo.gl/kjVydz) with a cluster extent of *p* < .05 corrected for multiple comparisons across the whole brain. As a result, the cluster extent threshold for GLM2 was set at *k* = 351, with an initial selection threshold of *p* < .001.

### 2.8 Region of interest analysis

In addition to the whole‐brain analyses, we further defined two types of Regions of Interest (ROIs) related to the discrimination of pain expressions, and then, performed an ROI analysis. First, regions representing empathy for pain based on a meta‐analysis (Lamm et al., 2011) were selected, including the anterior mid‐cingulate cortex (aMCC, MNI peak: −2, 23, 40), the left anterior insula (lAI, MNI peak: −40, 22, 0), and the right anterior insula (rAI, MNI peak: 39, 23, −4). Spheres of 10 mm radius centered at each peak coordinate were created as ROI masks (Rütgen et al., 2015b). Second, regions from the significant activation of parametric modulators were identified: a significant cluster in the right visual association cortex (MNI peak: 18, −84, −12; Brodmann area 18, 19, and 37) was found to be positively correlated with pain intensity averaged across two sessions (i.e., naltrexone vs. placebo; contrast weight [0.5, 0.5]; see Figure 5a,b below). This visual ROI was orthogonal to the analyses that were subsequently performed.

Drug effects (i.e., naltrexone > placebo) were checked in terms of the mean activation in those ROIs (aMCC, lAI, and rAI), regardless of pain intensity. We did not perform this analysis on the visual ROI as the definition of this ROI implicitly contained pain intensity information. The mean signal values of each ROI were extracted with the REX toolbox (Massachusetts Institute of Technology, Cambridge, MA, USA). Three two‐tailed paired *t*‐tests on drug effects were conducted. Additionally, we investigated whether any drug effects occurred in all four ROIs as the pain intensity increased (i.e., drug effects of the parametric regressors). We first tested if there was a significant ROI activation of the parametric regressors averaged across the two sessions (i.e., naltrexone vs. placebo: contrast weight [0.5, 0.5]) after small volume correction (SVC). To achieve an estimate of the brain activity regarding each specific experimental condition, percent signal change (PSC) values were estimated and applied in the following analyses (Gläscher, 2009). As only the visual ROI passed SVC, we, respectively, extracted PSC values for each individual in the visual ROI on all seven pain intensities for both sessions, using the rfxplot toolbox (Gläscher, 2009; http://rfxplot.sourceforge.net/).

We then examined putative drug effects in the PSC values of each pain intensity with a LME model. The model was performed using the lme4 package (v 1.1‐21; https://cran.r‐project.org/web/packages/lme4/index.html) in R. The full model included drug (Naltrexone and Placebo), pain intensity (20%, 30%, 40%, 50%, 60%, 70%, and 80%), and their interaction as fixed factors and subject identity as a random intercept. As the interaction term was not significant, we removed it from the model and report results from the model which only included the main effects. Statistical significance was calculated with Satterthwaite approximation for degrees of freedom and set as *p* < .05.

## 3 RESULTS

### 3.1 Behavioral results

#### 3.1.1 Slope and PSE of sigmoid function

**FIGURE 2:**
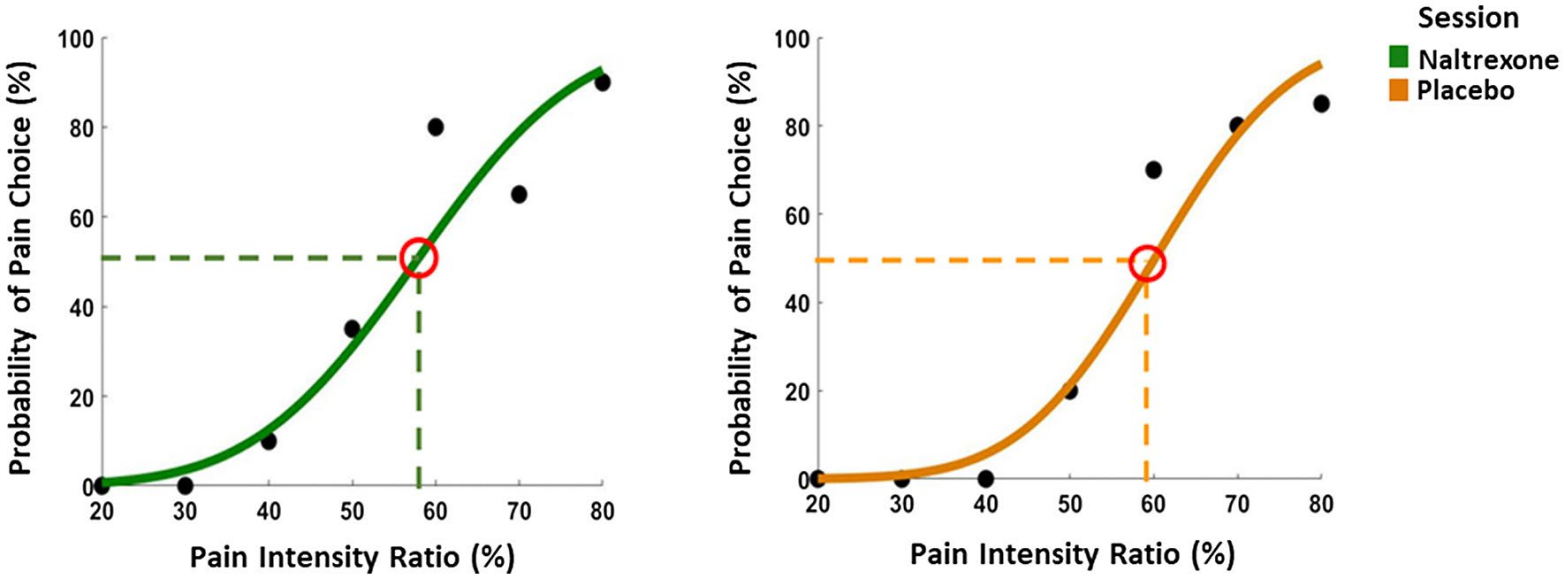
Example subject's Sigmoid function showing the relationship between pain intensity and pain response. For the shown subject's data, there is no difference in either the point of subjective equivalence (PSE), or the slopes in the naltrexone session (left panel) and the placebo session (right panel). PSE is indicated by the red circle in each plot, and the average slope of the curve generally represents how accurate when participants judged an expression as pain or disgust. The steeper the slope, the better the performance

In general, sigmoid functions showed good individual fit for both the naltrexone and the placebo sessions (see Figure 2 for an example subject's fitted curve). Paired *t*‐tests showed that there were no significant drug effects, neither in slope (*t*_41_ = 0.46, *p* = .65) nor in terms of the PSE values (*t*_41_ = 1.26, *p* = .22) of the fitted Sigmoid functions.

#### 3.1.2 LME model of pain choices

**FIGURE 3:**
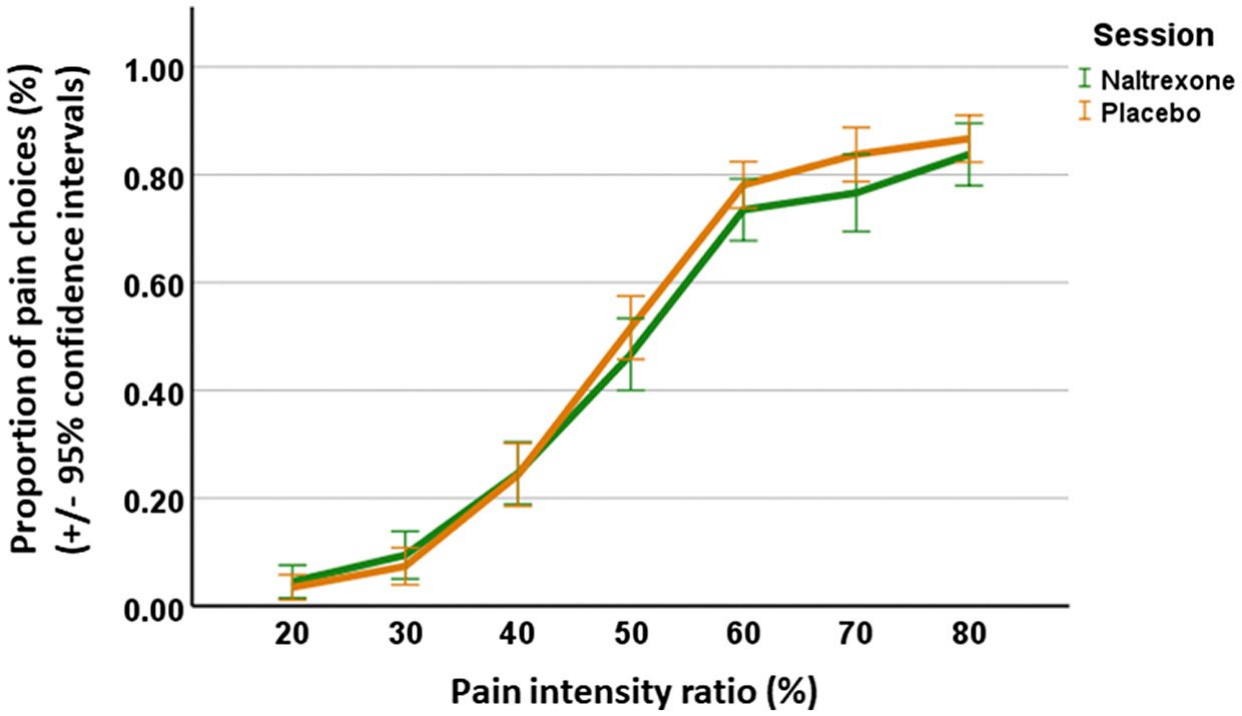
Effects of drug and pain intensity on responses of pain expressions. The proportion of judging an expression as pain at each pain intensity was illustrated for the naltrexone session (green) and the placebo session (orange). For the effect of drug, LME analysis indicated that the administration of naltrexone on average induced fewer pain choices compared with the placebo session. For the effect of pain intensity, in general, more pain choices were made as pain intensity increased. Error bars represent the 95% confidence interval

Results from the LME model (main effects only model; see Methods) for drug and intensity showed that the main effect of drug (*F*_1,488_ = 3.92, *β* = .47, *p* = .048) and intensity (*F*_6,489_ = 497.41, the smallest *β* = .80, *p* < .0001) were both significant. The post‐hoc Tukey test between different levels of pain intensities (across naltrexone and placebo sessions) showed that the majority of comparisons (except 30% vs. 20% pain, 70% vs. 60% pain, and 80% vs. 70% pain) were significant (Table S1). On average, participants made less frequent pain choices in the naltrexone session than in the placebo session, and generally (no interaction effect with drug) when pain intensity increased, participants showed an increasing probability of making a pain judgment (Figure 3).

#### 3.1.3 Comparison of side effects

The two‐tailed paired *t* test on the sum score of potential side effects of naltrexone between sessions remained nonsignificant (*t*_41_ = 1.59, *p* = .12). The two‐tailed Fisher's exact test on differences in reported nausea between sessions was not significant either (*p* = 1.00). Specifically, only two participants reported nausea in the naltrexone session and one reported nausea in the placebo session.

### 3.2 Imaging results

#### 3.2.1 Task manipulation check and parametric modulation

**FIGURE 4:**
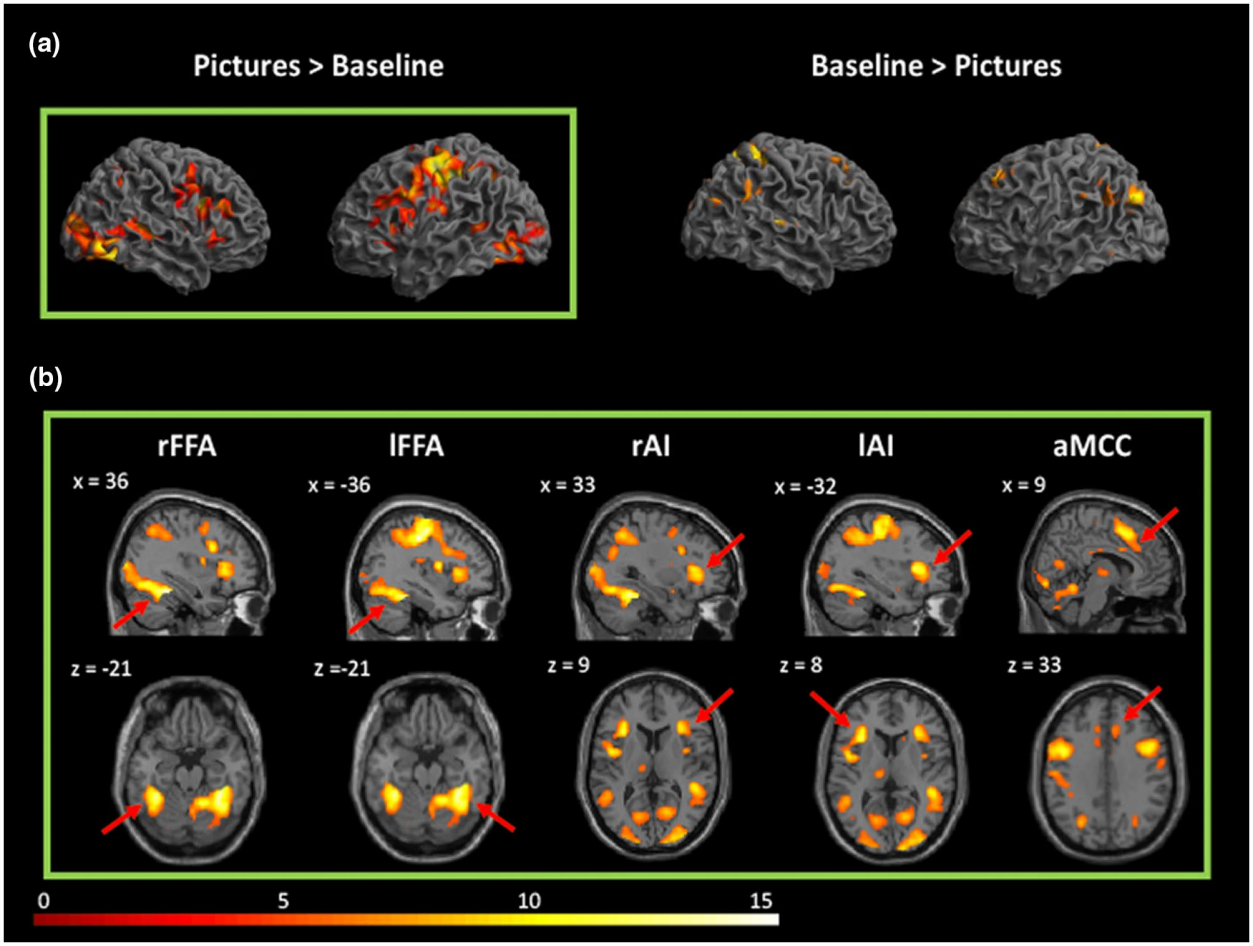
Neural correlates of the contrasts of pictures > baseline and baseline > pictures. (a) In the upper panel, pictures > baseline mainly revealed activation in left postcentral gyrus, right fusiform gyrus, left inferior frontal gyrus, left supplementary motor area, right angular gyrus, and right calcarine sulcus (*k* > 1,000); baseline > pictures, mainly activated right precuneus, left middle occipital cortex, left middle frontal cortex (*k* > 1,000). More extensive and stronger activity was generally detected when comparing pictures versus baseline than the reverse contrast. (b) In the lower panel, pictures > baseline showed that the paradigm led to significant activation in regions that we were interested in. aMCC, anterior mid‐cingulate cortex; lAI, left anterior insula; lFFA, left fusiform face area; rAI, right anterior insula; rFFA, right fusiform face area. Thresholded at voxel‐level FWE corrected *p* < .05

We performed two contrasts for the manipulation check: pictures > baseline, and the reverse contrast baseline > pictures. As expected, the brain network underlying perception of facial expressions was widely activated, including regions that we were interested in, namely, the FFA, anterior insula, and anterior mid‐cingulate cortex. See Figure 4 for a graphical display of the two contrasts, and Table 1 for a summary of all findings.

**TABLE 1:** Results of the manipulation check for contrasts pictures > baseline and baseline > pictures (*p* < .05 voxel‐level FWE‐corrected), in *MNI* space. Comparing pictures versus baseline revealed significantly stronger activation in right visual cortices. Besides, more brain areas and more voxels, in general, were activated with the contrast of pictures versus baseline compared to baseline versus pictures. These findings suggest that the task manipulation was successful. Region names were labeled with the AAL atlas

Region label	BA	Cluster size	*x*	*y*	*z*	*t* value
*Pictures > baseline*						
Postcentral_L	1	18,565	−38	−25	45	15.01
Fusiform_R	37	19,239	36	−43	−21	14.08
Frontal_Inf_Oper_R	44	7,770	44	11	24	13.38
Supp_Motor_Area_L	6	5,379	−4	8	54	13.03
Thalamus_L	50	903	−12	−21	2	9.79
Angular_R	39	2,238	33	−58	46	9.29
Calcarine_R	17	1,051	16	−66	9	8.79
Insula_R	13	189	38	−1	12	8.39
Cingulum_Mid_R	24	123	6	6	30	8.32
Temporal_Mid_L	21	526	−48	−48	8	7.82
Calcarine_L	17	660	−14	−70	9	7.81
Lingual_R	30	204	16	−36	0	6.97
Amygdala_R	34	20	33	2	−18	6.77
Thalamus_R	50	202	8	−13	2	6.73
Cerebellum_L	18	15	−8	−75	−44	6.28
Paracentral_L	1	80	−4	−30	60	6.09
Cuneus_R	19	14	15	−66	34	5.36
Temporal_Sup_R	22	2	52	−9	−9	5.16
*Baseline > pictures*						
ParaHippocampal_L	36	830	−34	−39	−10	10.61
Precuneus_R	7	6,835	4	−49	58	9.99
Temporal_Sup_R	41	423	59	−27	9	8.47
Occipital_Mid_L	39	1,539	−40	−76	30	8.17
Cuneus_L	18	603	−2	−96	18	8.15
Frontal_Mid_L	8	1,031	−22	24	46	7.99
Precuneus_L	23	822	−8	−54	9	7.9
Insula_R	13	362	42	−9	−4	7.23
Angular_R	39	589	57	−51	30	6.86
Frontal_Sup_R	8	712	26	30	50	6.79
Occipital_Mid_R	19	121	42	−76	26	6.63
Hippocampus_R	54	44	39	−24	−12	6.41
Frontal_Mid_L	6	78	−40	15	54	6.39
Frontal_Sup_Medial_L	10	378	−10	51	20	6.32
Frontal_Mid_R	9	25	29	51	39	6.19
Temporal_Inf_L	37	50	−58	−54	−14	6.13
Frontal_Sup_L	10	37	−24	63	26	6.12
Cerebelum_L	19	19	−30	−78	−39	5.66
Frontal_Sup_R	9	2	22	−10	69	5.36
SupraMarginal_R	40	9	48	−27	26	5.29
Occipital_Sup_R	19	18	24	−81	39	5.27

Abbreviations: BA, Brodmann area; L, left hemisphere; R, right hemisphere.

**FIGURE 5:**
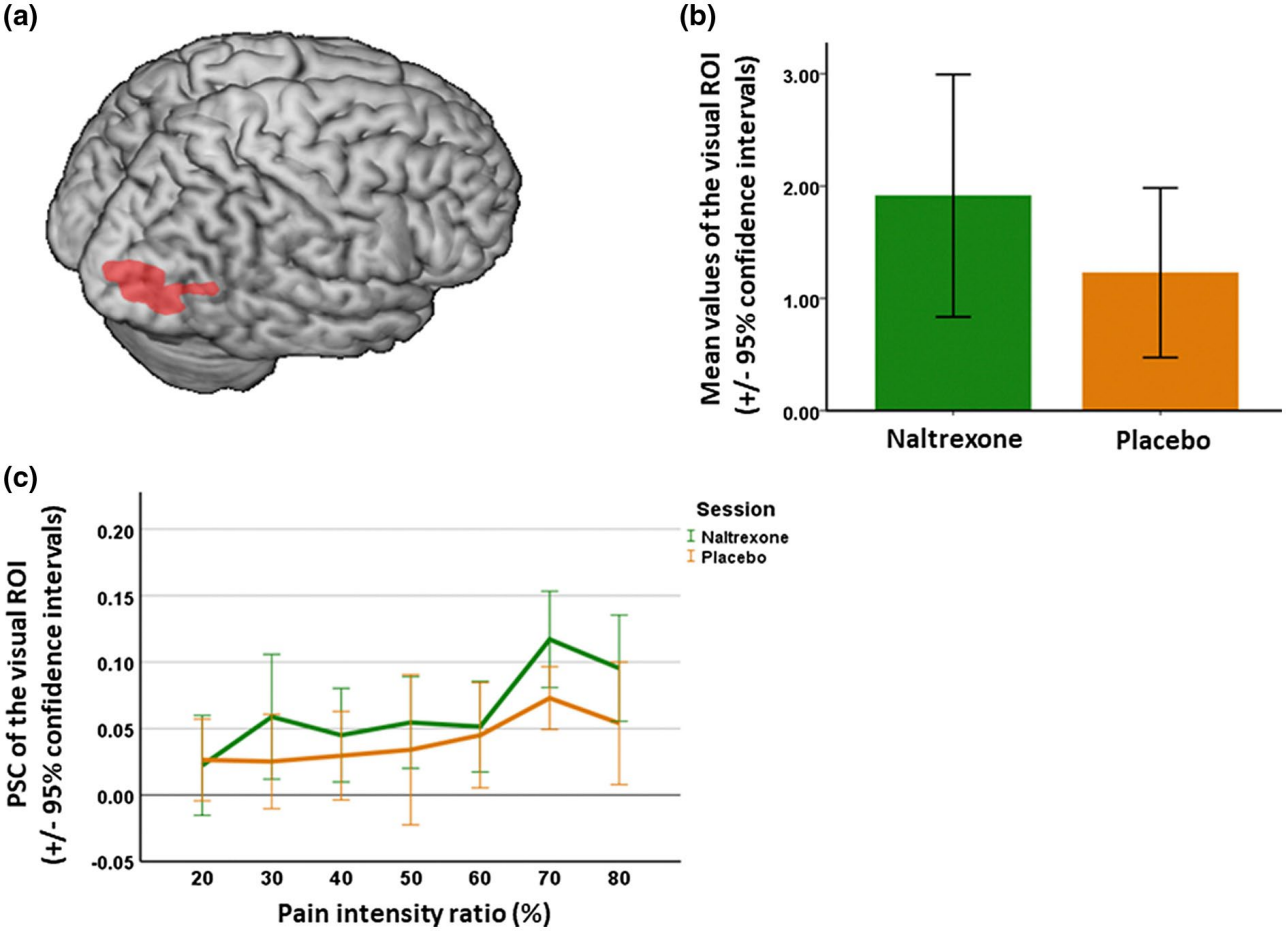
Neuroimaging findings of parametric activation in right visual cortex. (a) Neural correlates of parametric effects of pain intensity averaged across sessions. Activation was mainly localized to the right higher order visual cortex (MNI peak: 18, −84, −12), and encompassed what has been referred to as the fusiform face area (FFA). This activity was observed under the threshold of *p* < .05 cluster‐level FWE correction. (b) Mean activations of the parametric modulators in the visual ROI in the naltrexone session and the placebo session. Both sessions showed positive mean values of the parametric modulators in the visual ROI. This result indicates that as the intensity of expressed pain increased, activity in the ROI increases as well, in both sessions. (c) Effects of drug and pain intensity on PSC in the visual ROI. For the effect of drug, significant higher parametric modulation of the ROI activation on average was observed in the naltrexone session compared to the placebo session; for the effect of pain intensity, in general, high pain intensities (e.g., 60%, 70%, and 80%) showed increased activation in the visual ROI compared with low pain intensities (e.g., 20%, 30%, and 40%). Error bars show the 95% confidence interval

Within the parametric modulation model, the contrast on naltrexone versus placebo ([0.5, 0.5]) revealed a cluster in the right visual association cortex (MNI peak: 18, −84, −12), including what has been labeled in previous research as the FFA (e.g., MNI local peak: 26, −54, −14); see Figure 5a,b. In other words, this area showed a parametric increase of activation with increasing pain intensity, on average across both sessions.

#### 3.2.2 ROI results

We performed follow‐up ROI analyses on the defined ROIs. First, three paired *t*‐tests were performed on ROIs aMCC, lAI, and rAI to test whether there were significant drug effects on the means of seven pain intensity. None of the three regions showed significant difference between the naltrexone session and the placebo session (*t*_41_ = 0.10, −0.003, and −0.20, respectively, all *p* values >.86). Next, SVC was performed for all four ROIs to investigate whether there was significant parametric activation across the two sessions. Results showed that only the visual ROI passed the SVC (*p* < .0001, cluster‐level FWE‐corrected) with the initial threshold of *p* < .001, uncorrected. Therefore, the following LME analyses were only performed with the visual ROI.

#### 3.2.3 LME model of brain activation

Results showed that significant main effects of drug (*F*_1,484_ = 4.57, *β* = −.02*, p* = .03) and intensity (*F*_6,486_ = 3.48, the smallest *β* = .013, *p* = .002), see also Figure 5c. As for drug, activation (percent signal change) was on average higher in the naltrexone than in the placebo session. As for intensity, a Tukey post‐hoc showed that, when comparing higher pain intensity with lower pain intensity conditions, all *t* values were positive, and among these comparisons, significant higher PSC values were achieved in the comparisons of 70% versus 20% pain and 70% versus 40% pain (*t*_485_ = 3.89, *p* = .002; *t*_488_ = 3.14, *p* = .03), and trends in the same direction were observed in the comparisons of 70% versus 30% pain and 80% versus 20% pain (*t*_487_ = 2.85, *p* = .07; *t*_485_ = 2.80, *p* = .08). Overall, the results thus indicate that on average there was a higher activation in the visual ROI in the naltrexone session compared with the placebo session, and that the higher pain intensities in general, and irrespective of drug, showed stronger activation than the lower pain intensities.

### 3.3 Association between visual neural activity and pain choices

**FIGURE 6:**
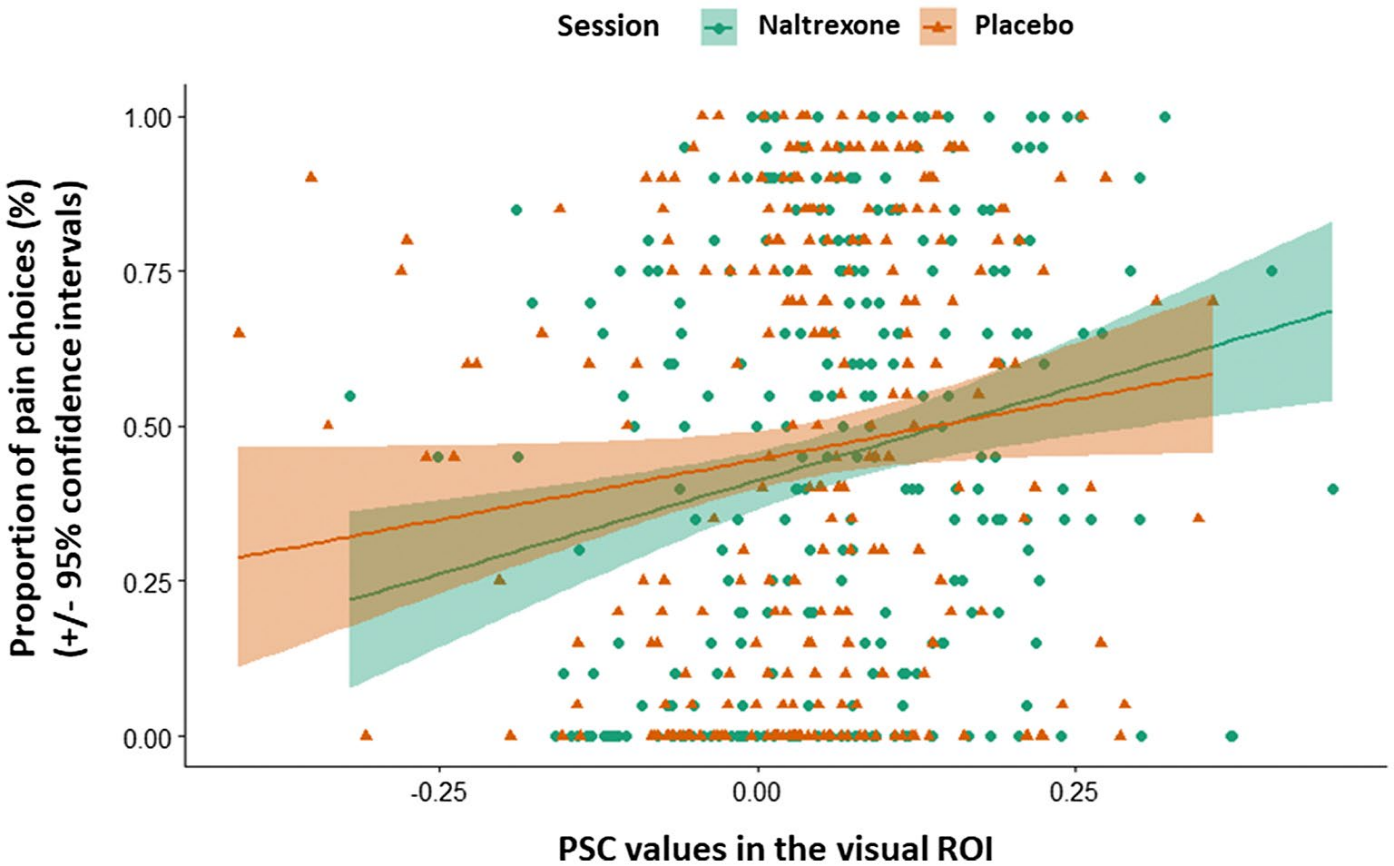
Association between brain and behavior. LME regressions of PSC values in the visual ROI predicting the proportion of pain choices. Each data point represents an individual's proportion of pain choices and its corresponding PSC activation at a certain pain intensity in the naltrexone session (green) and the placebo session (orange). Shaded regions indicate the 95% confidence interval

Two LME regression analyses were conducted to explore whether the neural activation in the visual ROI could predict behavioral responses in the naltrexone session and the placebo session, respectively. Results showed that in the naltrexone session, PSC in the visual ROI explained 4% of the variation in the proportion of pain choices, *R* ^2^ = .04, *p* = .001; and in the placebo session, PSC in the visual ROI explained 2% of the variation in the proportion of pain choices, *R* ^2^ = .02, *p* = .047. (Figure 6). The one‐sample *t* test of Fisher's *z* scores showed there was no statistical difference in *R* ^2^ between the two sessions (*p* = .36). This implies that the visual ROI on average explained 3% of the variance of the behavioral choice, and that the naltrexone and placebo sessions did not differ in their brain‐behavior predictions.

## 4 DISCUSSION

The present study aimed to examine whether the opioid system influences the perception of pain expressions, using an emotion discrimination task. A double‐blind cross‐over within‐subject design was applied, to investigate whether administration of the opioid antagonist naltrexone affected participants' judgments on whether a facial expression showed pain, and with what kind of neural activation this was associated. In brief, the results indicate that naltrexone decreased participants' discrimination on pain expressions, and more pain choices were generally made for higher pain intensities. The disturbance of the endogenous opioid system by naltrexone induced more activation in the right visual association cortex including FFA, and higher visual activity was detected with the high pain intensities (i.e., 70% and 80%) compared with the low pain intensities (i.e., 20%, 30%, and 40%).

We find that naltrexone decreased the probability for participants to judge an expression as pain. This finding provides evidence of the effects of the opioid antagonist on the discrimination of facial pain expression, suggesting a lower sensitivity to painful facial expressions resulting from the decrease in opioid system activity. Studies have demonstrated a reduction in seeking certain social cues under antagonism of the opioid system by naltrexone (Chelnokova et al., 2016; Wardle et al., 2016). The evolutionary meaning of pain is believed to work as a cue to aversive stimuli that constitute a potential threat to the individual (Broom, 2001; Kavaliers, 1988). In this respect, pain and in particular expressions that can be perceived by others also has an important social and communicative function, allowing the person in pain to signal that they are in need of help, apart from signaling potential dangers and threat to others. Thus, seeing others in pain is likely to induce empathy and concern, and to increase the intention of showing prosocial behaviors, including the provision of psychological comfort and concrete helping behaviors (Goubert et al., 2005; Hein et al., 2011; Masten et al., 2011; van der Meulen et al., 2016). In this respect, it is important to note that the opioid system has been linked to different facets of prosociality. For instance, release of endogenous opioids has been associated with social bonding, attachment, and empathy (Machin & Dunbar, 2011; Nummenmaa & Karjalainen, 2018; Nummenmaa & Tuominen, 2018; Rütgen et al., 2015b, 2018). Therefore, the decreases in discriminating others' facial expressions as showing pain under opioidergic blockade might suggest an attenuation in the sensitivity of pain expression perception, which ultimately may result in a decrease in the social‐affective link between persons, and a corresponding reduction in prosociality. This needs to remain a speculation, and the current data are not conclusive in this respect, as activation in the insular and cingulate cortex, i.e., areas previously linked to empathy and prosocial concern, were not affected by the opioid antagonist.

The observation that higher order visual cortex was the only area that showed effects of naltrexone thus poses the question what kind of processes were affected by the opioid system in the current study. The LME results show that stronger blood‐oxygen‐level‐dependent (BOLD) signals of the parameter pain intensity in the visual ROI were detected in the naltrexone session compared with the placebo session, indicating a significant distinction in the visual activity specifically related to discriminating pain expressions. One possibility is that the increased visual activation under naltrexone might reflect a compensatory effect in coping with the reduced visual sensitivity to pain expressions. Previous studies have stated that the opioid system was engaged in maintaining visual perceptual processing, for example, visual attention (Chelnokova et al., 2016; Dalley et al., 2005). In this study, the normal visual perceptual processing was disturbed by naltrexone. As a consequence, the visual sensitivity to the facial expressions of pain was affected. In order to recover or compensate for the blunting of visual sensitivity, there may have been an increase in activity in the visual cortex (possibly of neurons that do not use opioidergic neurotransmission) in an attempt to overcome the reduced visual sensitivity. This idea of the visual compensatory effect is supported by research on old adults (Riis et al., 2008) and patients with Alzheimer's disease (Bokde et al., 2009). However, it should be noted that this compensatory effect is merely a fine adjustment and cannot reverse the whole activation pattern in the visual cortex.

The results of how the visual ROI activation predicted behavioral responses demonstrate that the visual activation detected in this study is necessarily associated with discriminating pain expression of varied intensities. It showed that irrespective of the pharmacological manipulation, visual activation in that area was always positively correlated with and predicted, though with a rather low effect size, the increased intensities of pain expression. The location of the visual ROI is centered in the extrastriate cortex (i.e., V2, V3, and V4), and extends to the middle fusiform gyrus. This subregion of the fusiform gyrus has been repeatedly referred to as the “FFA” (Dubois et al., 1999; Halgren et al., 1999; Haxby et al., 1999; Kanwisher et al., 1997; McCarthy et al., 1997; Tong & Nakayama, 1999). Studies have robustly revealed that the FFA exhibits a stronger activation to faces rather than nonface stimuli (Halgren et al., 1999; Haxby et al., 1999; Kanwisher et al., 1997; McCarthy et al., 1997). Even though it has been suggested that FFA is an area linked to perceptual identification of the face, some studies have also indicated its involvement in processing facial expressions (Fox et al., 2009; Ganel et al., 2005). On top of that, the function of processing facial information in FFA has been found to exhibit a right hemisphere dominance (Barton et al., 2002; Fox et al., 2009; Ganel et al., 2005; Haxby et al., 1999; Rossion, Caldara, et al., 2003; Rossion et al., 2003). This is confirmed in our study for that only activation in the right FFA instead of the left was observed when facial expressions were perceived with increased pain intensity. In accordance with previous studies, our findings verify that the right FFA not only gets engaged in general facial recognition, but also modulates perception on facial expression features such as the intensity of pain expressions (Calder & Young, 2005; Eichmann et al., 2008; Loughead et al., 2008). Furthermore, this activity found in the right FFA was modulated by the opioid system, suggesting an underlying opioidergic mechanism engaged in the facial processing of emotional expressions via the ventral stream.

As pain intensities increased, we did not find significant parametric increases in activity of the anterior insula and the anterior mid‐cingulate cortex. This could be related to the aversive nature of both pain and disgust. However, it is important to note that our manipulation check analyses showed significant activity in the bilateral anterior insular cortex and the anterior mid‐cingulate cortex when comparing the task with baseline, as expected. Therefore, the absence of parametric modulation in these regions might imply naltrexone had no unique modulatory effect on the affective components of perceiving pain expressions.

We would also like to address the potential clinical implications of our work, which may have significant meaning with respect to the diagnosis and therapeutic interventions of pain. First, patients with chronic pain, especially those who were high in fear of (re)injury, showed strong attention bias toward painful facial expressions (Khatibi et al., 2009). Furthermore, participants with high catastrophizing personality were found to exhibit longer gaze duration for both pain and neutral expressions (Vervoort et al., 2013). Observation of others' pain expressions, however, has been detected to increase pain perception in self (Khatibi et al., 2014, 2015; Mailhot et al., 2012; Reicherts et al., 2013; Vachon‐Presseau et al., 2012). It has thus been argued that the facilitation of pain perception induced by vicarious pain might be modulated by top‐down attentional processes (Khatibi et al., 2014). In terms of our findings, it indicates that when administrating opioidergic analgesics (e.g., painkillers) to diminish patients' pain, the analgesic effect might be counteracted by the enhanced attention toward others' pain expressions. Second, our findings also raise the issue that applying opioidergic analgesics might affect how accurately and efficiently we detect pain in others. More specifically, the reduction in our capacity or efficiency to accurately identify pain‐related expressions may affect the diagnosis and treatment of patients' pain by medical personnel under the influence of (opioid) painkillers. This is especially important considering the current “opioid endemic,” with about 11.5 million people in the USA alone showing a misuse of prescription opioids in 2016 (National Center for Health Statistics, 2017; Substance Abuse & Mental Health Services Administration, 2017).

The endogenous opioid system is engaged in modulating many affective and cognitive functions, such as visual perception, emotion, and reward (Chelnokova et al., 2016; Colasanti et al., 2012; Dalley et al., 2005; Koepp et al., 2009; Liberzon et al., 2002; Rütgen et al., 2015b). Studying the neurochemical underpinnings of emotion perception may also have important implications in understanding the experience and processes of empathy. A recent framework of empathy considers that emotion identification and affect sharing are two separate processes that independently contribute to empathic responses (Coll et al., 2017). While emotion identification in this framework is closely related to but not synonymous with emotion discrimination, failure to clarify the possibly distinct effects of emotion discrimination and affect sharing might lead to inaccurate characterization of the experience of empathy. The findings from the present study are thus particularly relevant for the further development and validation of this framework. They suggest that the opioid system not only plays a role in higher order affective processes underpinning empathy, and in particular affect sharing, but that causally manipulating opioidergic activity also modulates the perception and judgment of pain in others. This, we hope, will inspire further exploration of the relationship between the opioid system and socio‐perceptual processes, and how they inform higher level processes and aspects of the multi‐faceted experience of empathy. However, in making these connections, it needs to be considered that the present study used an opioid antagonist to investigate the role of the opioidergic activity on pain discrimination. Interestingly, our findings seem at odds with the predictions and analyses reported in Coll et al., which would seem to suggest an increase rather than a decrease in pain discrimination with reduced opioid activity. Future studies with opioid agonists (Chelnokova et al., 2016). or other types of painkillers (Mischkowski et al., 2016) are thus needed to verify the possible links between analgesics and pain perception, in both self and others.

Finally, some limitations of this study deserve discussion. First, the different degrees of pain/disgust in the stimuli were determined by the morphing software, and may thus not accurately reflect the subjective experience of these degrees by study participants. Nevertheless, this method is frequently used in studies on emotion identification (Averbeck et al., 2011; Wells et al., 2016; Young et al., 1997), and our behavioral results suggest that the morphed degrees used in our study corresponded approximately with subjective experience. Second, variable inter‐trial intervals could be implemented between viewing pictures and the judgment phase in future studies, though this had little influence on our current findings due to parametric analysis approach and the documented quasi‐absence of multicollinearity (*r* = −.03). Third, naltrexone was suggested to be associated with increased attention to the negative valence of stimuli in general (Meier et al., 2016; Murray et al., 2014). Despite of the main focus of the current study being pain, further experiments are thus required to carefully test whether naltrexone similarly induces increased attention to both pain and disgust expressions and whether this general effect affects our current results. However, given that we detected differential effects for pain and disgust, we can rather safely say that in those aspects the drug had selective effects; this is, however, not to say that in other domains, additional effects could have been detected but were occluded due to a lack of selectivity associated with opioid blockade.

In conclusion, the behavioral and neural findings of this psychopharmacological fMRI study shed light on a causal role of the opioid system in the discrimination of painful facial expressions, paving the way for further exploration of clinical implications in the domains of pain diagnosis and treatment, on the one hand, and future research on the relationship between basic socio‐perceptual processing and empathy, on the other.

## CONFLICT OF INTEREST

The authors declare no competing financial interests.

## AUTHOR CONTRIBUTIONS

**Yili Zhao:** Conceptualization; Data curation; Formal analysis; Funding acquisition; Investigation; Methodology; Project administration; Resources; Software; Validation; Visualization; Writing‐original draft; Writing‐review & editing. **Markus Rütgen:** Conceptualization; Formal analysis; Investigation; Methodology; Project administration; Resources; Software; Validation; Writing‐original draft; Writing‐review & editing. **Lei Zhang:** Formal analysis; Methodology; Resources; Software; Validation; Writing‐original draft; Writing‐review & editing. **Claus Lamm:** Conceptualization; Formal analysis; Funding acquisition; Methodology; Resources; Supervision; Validation; Writing‐review & editing.

## Supporting information

Supplementary Material
