# Supplementary material for: Pharmacological fMRI provides evidence for opioidergic modulation of discrimination of facial pain expressions
Source: Psychophysiology. 2020 Nov 3;58(2):e13717. doi: 10.1111/psyp.13717 (PMC7816233; doi:10.1111/psyp.13717)
Supplement: Supplementary file 1 — Supplementary Material [file PSYP-58-e13717-s001.docx]

***Supplementary Table 1.*** Post-hoc Tukey’s test: *t* values of the pairwise comparisons between pain intensities across naltrexone and placebo sessions

| **Pain intensity** | **20%** | **30%** | **40%** | **50%** | **60%** | **70%** |
| --- | --- | --- | --- | --- | --- | --- |
| **20%** |  |  |  |  |  |  |
| **30%** | 1.85 |  |  |  |  |  |
| **40%** | 9.59*** | 7.46*** |  |  |  |  |
| **50%** | 20.37*** | 18.04*** | 10.89*** |  |  |  |
| **60%** | 33.07*** | 30.30*** | 23.09*** | 11.59*** |  |  |
| **70%** | 34.68*** | 31.91*** | 24.81*** | 13.44*** | 2.08 |  |
| **80%** | 36.69*** | 33.86*** | 26.86*** | 15.41*** | 4.17** | 2.06 |

*p* < .05*, *p* < .001**, *p* < .0001**
